# Supplementary material for: Limited geographic genetic structure detected in a widespread Palearctic corvid, Nucifraga caryocatactes
Source: PeerJ. 2014 Jun 17;2:e371. doi: 10.7717/peerj.371 (PMC4081298; doi:10.7717/peerj.371)
Supplement: Table S1 — Eurasian nutcracker (Nucifraga caryocatactes) sample codes, geographic location, voucher number from Burke Museum of Natural History and Culture – University of Washington, and GenBank accession numbers. [file peerj-02-371-s001.pdf]

Supplemental Table S1. Eurasian nutcracker (*Nucifraga caryocatactes*) sample codes, geographic location, voucher number from Burke Museum of Natural History and Culture – University of Washington, and GenBank accession numbers.

Sample	Geographic Location	Voucher	GenBank
BK001	Badzhal'skiy Khrebet, Khabarovskiy Kray, Russia	47356, SVD 262	
BK002	Badzhal'skiy Khrebet, Khabarovskiy Kray, Russia	47357, SVD 263	
BK003	Badzhal'skiy Khrebet, Khabarovskiy Kray, Russia	46908, BKS 961	
GA001	Gorno-Altaysk, Altai Republic, Russia	46296, CDS 4858	
GA002	Gorno-Altaysk, Altai Republic, Russia	46293, CDS 4855	
GA003	Gorno-Altaysk, Altai Republic, Russia	46286, CDS 4848	
GA004	Gorno-Altaysk, Altai Republic, Russia	46285, CDS 4847	
KA001	Koryaki, Kamchatka, Russia	44307, JMB 1147	
KA002	Milkovo, Kamchatka, Russia	44356, JMB 1198	
KA003	Milkovo, Kamchatka, Russia	44357, JMB 1199	
KA004	Milkovo, Kamchatka, Russia	44359, JMB 1201	
KA005	Milkovo, Kamchatka, Russia	44365, JMB 1210	
KA006	Milkovo, Kamchatka, Russia	44072, CSW 4670	
KD001	Khamar-Daban weather station, Slyudyanka, Irkutsk Oblast, Russia	51815, SVD 612	
KD002	Khamar-Daban weather station, Slyudyanka, Irkutsk Oblast, Russia	51817, SVD 614	
KD003	Khamar-Daban weather station, Slyudyanka, Irkutsk Oblast, Russia	51818, SVD 615	
KD004	Khamar-Daban weather station, Slyudyanka, Irkutsk Oblast, Russia	51826, SVD 623	
KD005	Khamar-Daban weather station, Slyudyanka, Irkutsk Oblast, Russia	51841, SVD 638	
MA001	Snezhnaya Dolina, Ol'skiy Rayon, Magadanskaya Oblast, Russia	51577, SVD 357	
MA002	Snezhnaya Dolina, Ol'skiy Rayon, Magadanskaya Oblast, Russia	51549, SVD 329	
MA003	mouth of Oroholyndja River, Magadan, Magadanskaya Oblast, Russia	44100, DAB 41	
MA004	mouth of Oroholyndja River, Magadan, Magadanskaya Oblast, Russia	44425, SAR 6050	
MA005	mouth of Oroholyndja River, Magadan, Magadanskaya Oblast, Russia	44400, JMB 987	
MA006	mouth of Oroholyndja River, Magadan, Magadanskaya Oblast, Russia	43849, CSW 4376	
MA007	mouth of Oroholyndja River, Magadan, Magadanskaya Oblast, Russia	43817, CSW 4344	
MA008	mouth of Oroholyndja River, Magadan, Magadanskaya Oblast, Russia	44192, JMB 1016	
OK001	Kyzyl, Ovyurskiy Kozhuun, Tuva Republic, Russia	66384, NAM 213	
OR001	headwaters of Ola River, Magadanskaya Oblast, Russia	44199, JMB 1024	
OR002	headwaters of Ola River, Magadanskaya Oblast, Russia	44198, JMB 1023	
PK001	Khrebet Siniy, Arsen'yev, Anuchinskiy Rayon, Primorskiy Kray, Russia	75252, RYA 551	
PK002	Khrebet Siniy, Arsen'yev, Anuchinskiy Rayon, Primorskiy Kray, Russia	75160, RYA 459	

PK003	Khrebet Siniy, Arsen'yev, Anuchinskiy Rayon, Primorskiy Kray, Russia	75159, RYA 458
PK004	Khrebet Siniy, Arsen'yev, Anuchinskiy Rayon, Primorskiy Kray, Russia	75248, RYA 547
PK005	Khrebet Siniy, Arsen'yev, Anuchinskiy Rayon, Primorskiy Kray, Russia	75249, RYA 548
PK006	Khrebet Siniy, Arsen'yev, Anuchinskiy Rayon, Primorskiy Kray, Russia	75250, RYA 549
PK007	Khrebet Siniy, Arsen'yev, Anuchinskiy Rayon, Primorskiy Kray, Russia	75130, RYA 427
PK008	Khrebet Siniy, Arsen'yev, Anuchinskiy Rayon, Primorskiy Kray, Russia	75161, RYA 460
PK009	Khrebet Siniy, Arsen'yev, Anuchinskiy Rayon, Primorskiy Kray, Russia	75200, RYA 499
PK010	Khrebet Siniy, Arsen'yev, Anuchinskiy Rayon, Primorskiy Kray, Russia	75251, RYA 550
PK011	Khrebet Siniy, Arsen'yev, Anuchinskiy Rayon, Primorskiy Kray, Russia	75129, RYA 426
SO001	Sakhalinskaya Oblast, Russia	47409, SVD 317
SO002	Sakhalinskaya Oblast, Russia	47410, SVD 318
SO003	Sakhalinskaya Oblast, Russia	47412, SVD 320
SO004	Sakhalinskaya Oblast, Russia	47598, VM 283b
SO005	Sakhalinskaya Oblast, Russia	47600, VM 284b
TO001	Vengoyakha river, Noyabr'sk, Yamalo-Nenetskiy Avtonomnyi Okrug, Tyumenskaya Oblast, Russia	56723, CSW 5577
TO002	Vengoyakha river, Noyabr'sk, Yamalo-Nenetskiy Avtonomnyi Okrug, Tyumenskaya Oblast, Russia	56746, CSW 5601
TO003	Vengoyakha river, Noyabr'sk, Yamalo-Nenetskiy Avtonomnyi Okrug, Tyumenskaya Oblast, Russia	56770, CSW 5626
TO004	Vengoyakha river, Noyabr'sk, Yamalo-Nenetskiy Avtonomnyi Okrug, Tyumenskaya Oblast, Russia	56792, CSW 5649
TO005	Vengoyakha river, Noyabr'sk, Yamalo-Nenetskiy Avtonomnyi Okrug, Tyumenskaya Oblast, Russia	56964, SVD 1198
TO006	Vengoyakha river, Noyabr'sk, Yamalo-Nenetskiy Avtonomnyi Okrug, Tyumenskaya Oblast, Russia	56972, SVD 1206
TO007	Vengoyakha river, Noyabr'sk, Yamalo-Nenetskiy Avtonomnyi Okrug, Tyumenskaya Oblast, Russia	56782, CSW 5639
TU001	Turuka, Ust-Kut, Ust-Kutskiy Rayon, Irkutsk Oblast, Russia	73336, JML 322
TU002	Turuka, Ust-Kut, Ust-Kutskiy Rayon, Irkutsk Oblast, Russia	73348, JML 334
TU003	Turuka, Ust-Kut, Ust-Kutskiy Rayon, Irkutsk Oblast, Russia	73681, VGR 451
TU004	Turuka, Ust-Kut, Ust-Kutskiy Rayon, Irkutsk Oblast, Russia	73478, RCF 2288
TU005	Turuka, Ust-Kut, Ust-Kutskiy Rayon, Irkutsk Oblast, Russia	73485, RCF 2295
UL001	Ulaanbaatar, Töv Aymag, Mongolia	59995, CSW 5965
UL002	Ulaanbaatar, Töv Aymag, Mongolia	60165, DAB 2728
UL003	Ulaanbaatar, Töv Aymag, Mongolia	60166, DAB 2729
UL004	Ulaanbaatar, Töv Aymag, Mongolia	60167, DAB 2730
UL005	Ulaanbaatar, Töv Aymag, Mongolia	60168, DAB 2731
